# Transgenic expression of the HERV-W envelope protein leads to polarized glial cell populations and a neurodegenerative environment

**DOI:** 10.1073/pnas.2308187120

**Published:** 2023-09-11

**Authors:** Joel Gruchot, Isabel Lewen, Michael Dietrich, Laura Reiche, Mustafa Sindi, Christina Hecker, Felisa Herrero, Benjamin Charvet, Ulrike Weber-Stadlbauer, Hans-Peter Hartung, Philipp Albrecht, Hervé Perron, Urs Meyer, Patrick Küry

**Affiliations:** ^a^Department of Neurology, Medical Faculty, Heinrich-Heine-University Düsseldorf, 40225 Düsseldorf, Germany; ^b^Institute of Veterinary Pharmacology and Toxicology, University of Zürich-Vetsuisse, CH-8057 Zürich, Switzerland; ^c^GeNeuro Innovation, 69008 Lyon, France; ^d^Neuroscience Center Zurich, University of Zürich and ETH Zürich, CH-8057 Zürich, Switzerland; ^e^Brain and Mind Center, University of Sydney, NSW 2050 Sydney, Australia; ^f^Department of Neurology, Palacky University Olomouc, 77146 Olomouc, Czech Republic; ^g^Department of Neurology, University of Bern, CH-3010 Bern, Switzerland

**Keywords:** endogenous retrovirus, multiple sclerosis, neurodegeneration, myelin repair, glia

## Abstract

The human endogenous retrovirus type W (HERV-W) has been identified and repeatedly confirmed as human-specific pathogenic entity affecting many cell types in multiple sclerosis (MS). Our recent contributions revealed the encoded envelope (ENV) protein to disturb myelin repair by interfering with oligodendroglial precursor differentiation and by polarizing microglial cells toward an axon-damage phenotype. Indirect proof of ENV’s antiregenerative and degenerative activities has been gathered recently in clinical trials using a neutralizing anti-ENV therapeutic antibody. Yet direct proof of its mode of action can only be presented here based on transgenic ENV expression in mice. Upon demyelination, we observed myelin repair deficits, neurotoxic microglia and astroglia, and increased axon degeneration. Experimental autoimmune encephalomyelitis activity progressed faster in mutant mice equally accompanied by activated glial cells. This study therefore provides direct evidence on HERV-W ENV’s contribution to the overall negative impact of this activated viral entity in MS.

## Significance

Although neurodegeneration is a hallmark of multiple sclerosis (MS), its progression is still not fully understood. However, it is the major factor leading to clinical disability that still cannot be addressed therapeutically. With the here presented study, we provide direct evidence that HERV-W (human endogenous retrovirus type W) ENV (envelope) expression results in multiple glial cell deteriorations and accompanied neuropathology in vivo. These data therefore suggest that activation of this endogenous retroviral element is indeed causally contributing to MS. Our findings will therefore help understand the molecular and cellular processes being modulated by the currently clinically tested anti-HERV-W neutralization strategy and will support the development of this approach into clinical therapy.

Multiple sclerosis (MS) is a demyelinating disease of the central nervous system (CNS) of still unknown etiology. This disease is primarily characterized by peripheral immune cell infiltration, focal inflammation, as well as the loss of oligodendrocytes and myelin sheaths leading to white and gray matter lesions. During disease progression, immune cell infiltration ceases and neurodegeneration predominates, leading to irreversible sensory, motor, and cognitive deficits (1). Besides peripheral immune cells, brain resident microglial and astroglial cells are also involved in the disease process particularly in progressive stages as they were shown to adapt neurotoxic- and damage-associated profiles and phenotypes (2).

In 1989, an association between retroviral elements and MS was described based on the analysis of primary leptomeningeal cell cultures isolated from MS patients (3). While initially termed MS-associated retrovirus, it was later found to belong to the family of human endogenous retroviruses (HERVs) and referred to as HERV type W (4). Subsequent studies on HERV-W (human endogenous retrovirus type W) provided convincing evidence that activation and expression of this otherwise dormant sequences and the subsequent production of the encoded envelope (ENV) protein can exacerbate the immune response (5–7). It was then shown that HERV-W ENV RNA and protein levels are increased in the cerebrospinal fluid (CSF) and serum of MS patients (8–10). In MS brains HERV-W ENV protein was found as acellular deposits and to be expressed by myeloid cells whereas HERV-W ENV-positive astroglial and lymphoid cells could also be detected (11, 12). Herpesviridae have been shown to activate dormant HERVs among them also the Epstein–Barr Virus (EBV) (13, 14), which was recently suggested to be a leading cause for MS (15), thus further corroborating a functional implication of HERV-W in the disease process.

Apart from immune and endothelial cell activation (5, 7, 16), our own research provided strong evidence that also myelin repair is impaired by HERV-W ENV (17, 18). Furthermore, a role in polarization of microglial cells toward a demyelination- and neurodegeneration-related phenotype was reported (11). Yet, owing to its human-specific origin these studies relied on histological assessments combined with functional experiments done ex vivo. Nevertheless, an implication of HERV-W in neurodegeneration and white matter repair was further supported by clinical trials on a HERV-W ENV neutralizing antibody termed temelimab as a significant reduction in brain atrophy levels as well as improved myelin integrity were observed in treated MS patients but also relied upon an indirect analysis, i.e., MRI (19).

We here report a role of HERV-W ENV in MS-related disease processes based on the analysis of a viable transgenic mouse line mimicking endogenous HERV-W ENV activity in the diseased CNS. Apart from exacerbated autoimmune activities, a strong negative impact on oligodendrogenesis and remyelination along with neurotoxic microglial and astroglial cell populations was observed.

## Results

### Transgenic HERV-W Env Expression Fosters Demyelination and Reduces Remyelination.

**Fig. 1.**
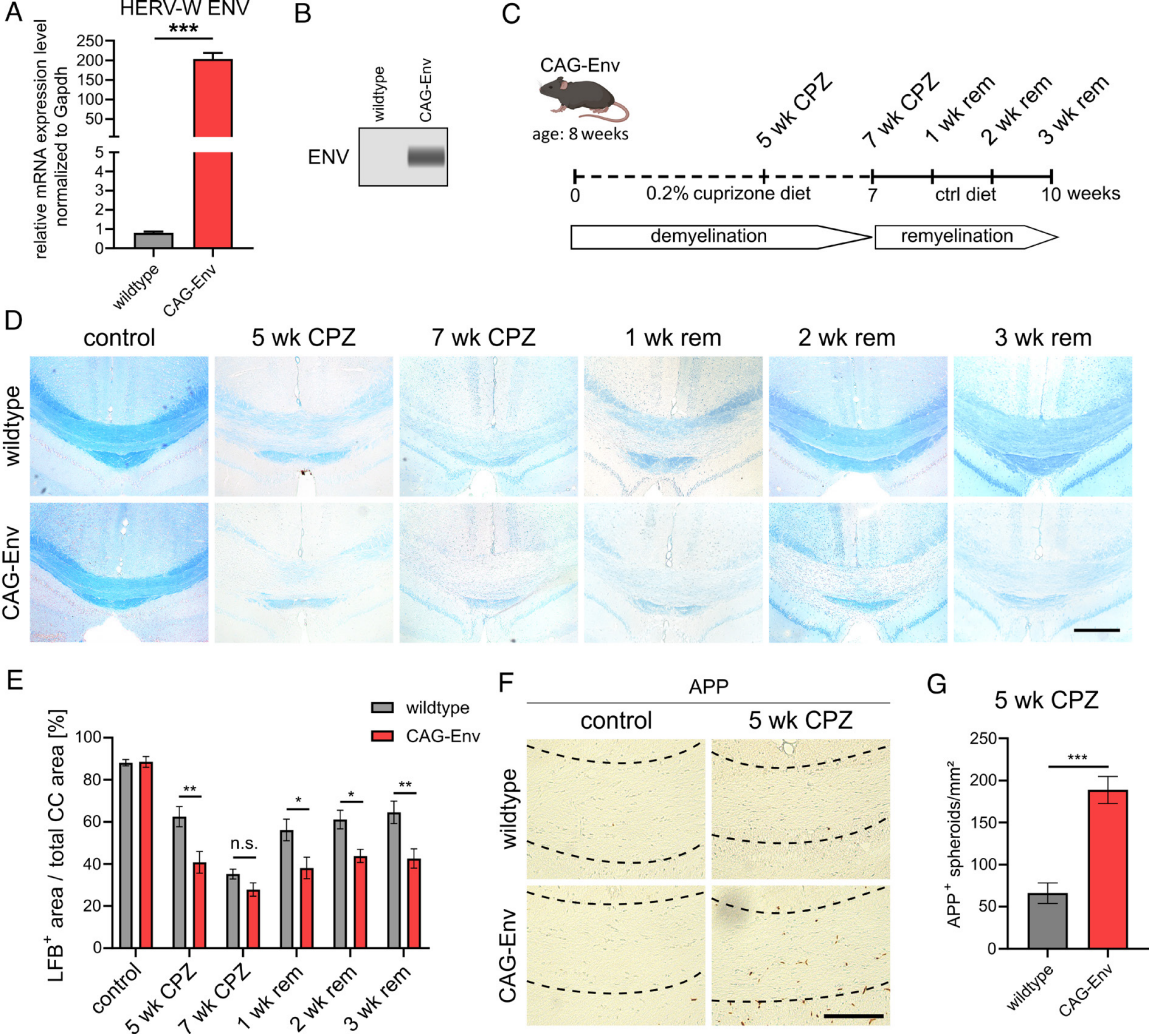
Transgenic HERV-W ENV expression fosters demyelination and alters remyelination upon CPZ treatment. (*A*) Determination of the relative ENV transcript levels normalized to Gapdh in whole-brain lysates of wt and CAG-Env transgenic mice. (*B*) Automated western blot analysis of whole-brain lysates detecting HERV-W ENV protein in transgenic mice. (*C*) Schematic presentation of CPZ demyelination and remyelination experiments. (*D*) Representative images of LFB-stained control and CPZ-challenged wt and transgenic tissue sections encompassing the caudal corpus callosum (corpus callosum) at time points 5 and 7 wk of CPZ treatment and at 1, 2, and 3 wk during remyelination. (*E*) Quantification of the percentage of LFB-positive, myelinated area of the corpus callosum. (*F*) Representative images of anti-APP stained wt and CAG-Env corpus callosum tissue sections at 5 wk of demyelination. (*G*) Quantification of APP-positive spheroid densities in the corpus callosum of wt and transgenic animals. Data are presented as mean values (*A* and *B*: n = 3; *E*–*H*: n = 6) ± SEM. Significance of HERV-W ENV mRNA levels as well as APP-positive spheroids were assessed by Student’s *t* test and the significance of the relative LFB-positive areas was accessed by 2-way ANOVA followed by Sidak’s post hoc. Data were considered as statistically significant (95% CI) at *P < 0.05, **P < 0.01, ***P < 0.001. n.s. = not significant. CC = corpus callosum. (Scale bar in *E*: 250 µm, scale bar in *G*: 100 µm.)

In our previous studies, the HERV-W ENV protein was shown to affect glial cells (18, 20) and in MS ENV expression was described by myeloid, astroglial and lymphoid cells, leading to acellular deposits of shed protein (12, 20). Such an expression pattern likely results from long-term activation scenarios but in light of the still missing information on HERV-W genomic integration sites and expression-driving sequences (20, 21) reveals to be difficult to mimic. In order to analyze the effects of endogenous ENV expression in a functional in vivo context, we used a previously established mouse model (22), in which the HERV-W ENV transgene (pV14; GenBank accession number: AF331500.1) is expressed under the control of the ubiquitous CAG promotor but is additionally regulated by its 3′ long terminal repeat sequence. This enabled a moderate expression of HERV-W ENV comparable to levels found in human tissue (10). To confirm ENV transcript and protein expression in the mouse brain RT-qPCR as well as automated western blot techniques were used (Fig. 1 *A* and *B*). Based on the reported hexameric extracellular appearance of the ENV protein and the resulting unique solubility and antigenic characteristics, particularly when using human-specific antibodies in a mouse background (23), the analysis via automated Simple Western technology revealed to be the essential. To analyze the effects of transgenic ENV expression on remyelination, we applied the well-established cuprizone (CPZ) model of demyelination (24–26). Transgenic and wt littermate mice (hemizygote males and homozygote females) were fed for 7 wk with 0.2% CPZ chow and subsequently switched to control diet to induce remyelination (Fig. 1*C*). Histological analysis was carried out at time points 5 and 7 wk of demyelination as well as at 1, 2, and 3 wk during remyelination. Luxol fast blue (LFB) staining of the caudal corpus callosum revealed that transgenic ENV protein increases/accelerates CPZ-mediated demyelination and further impedes remyelination of this brain structure (Fig. 1 *D* and *E*). Moreover, anti-amyloid precursor protein (APP) staining revealed increased densities of APP-positive spheroids in transgenic corpus callosum (Fig. 1 *F* and *G*), suggesting enhanced neurodegeneration in transgenic mice.

### Transgenic HERV-W ENV Expression Affects Oligodendroglial Cell Differentiation.

**Fig. 2.**
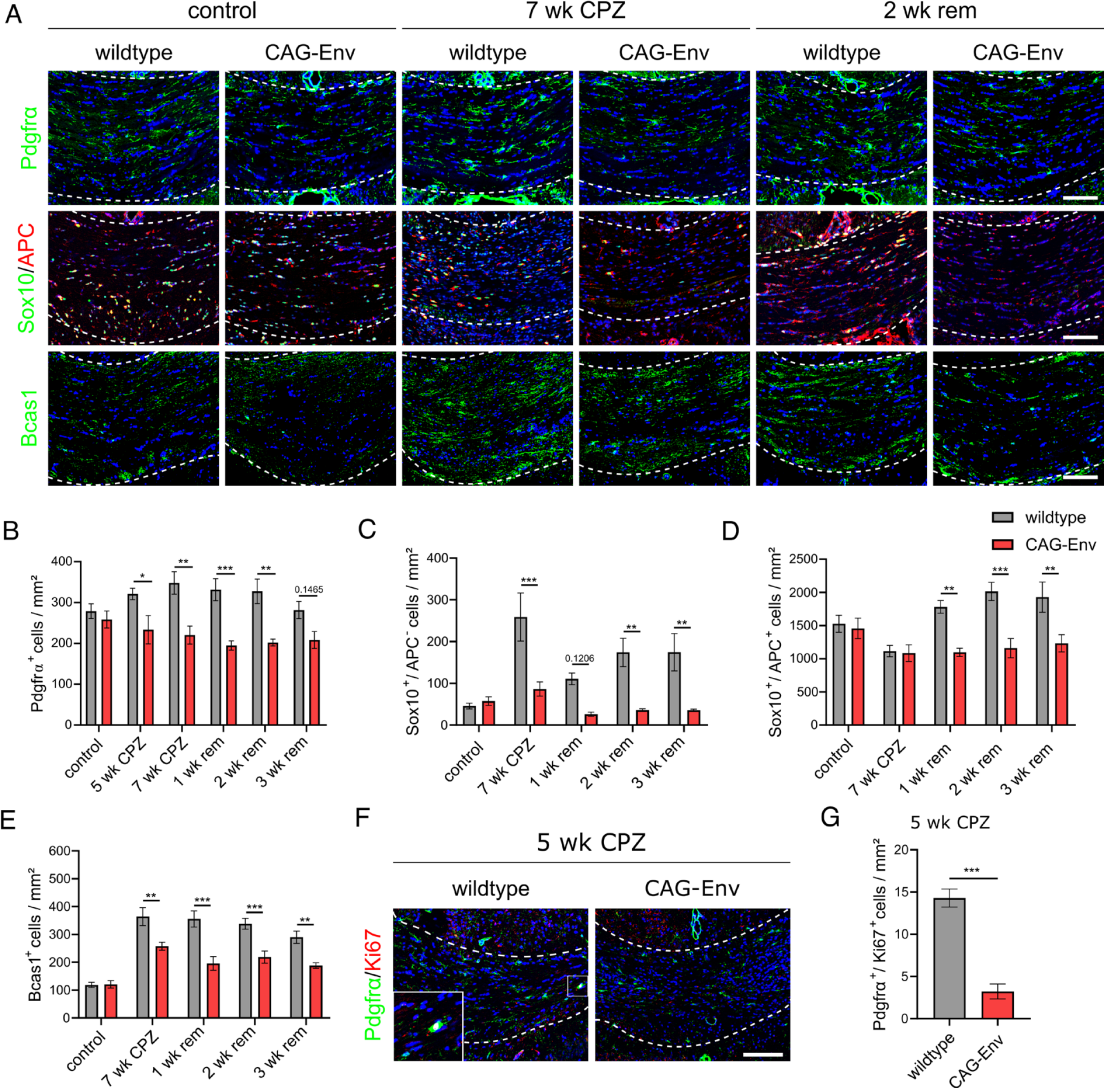
Transgenic HERV-W ENV expression affects oligodendroglial differentiation. (*A*) Representative immunohistochemical images of Pdgfrα-, Sox10/APC- and Bcas1- expression in unchallenged animals (wt and CAG-Env mice), after 7 wk of CPZ treatment and after 2 wk of CPZ withdrawal (2 wk rem). (*B*) Quantification of Pdgfrα-positive cell densities in the corpus callosum of control vs. CPZ-treated animals. (*C*) Quantification of Sox10-positive, APC-negative cell densities in the corpus callosum of control vs. CPZ-treated animals. (*D*) Quantification of Sox10/APC double-positive maturing oligodendroglial cell densities in the corpus callosum of control vs. CPZ-treated mice. (*E*) Quantification of Bcas1-positive myelinating oligodendrocyte densities in the corpus callosum of control vs. CPZ-treated animals. (*F*) Representative immunohistochemical pictures of Pdgfrα/Ki67-coexpressing cells in the corpus callosum of wt vs. CAG-Env mice at 5 wk of CPZ treatment. (*G*) Quantification of Ki67-positive proliferating OPCs in wt vs. CAG-Env corpus callosum tissues after 5 wk of CPZ diet. Data are presented as mean values (n = 6) ± SEM. Significance of Ki67-positive OPCs was analyzed by Student’s unpaired *t* test, whereas all other significances were accessed by 2-way ANOVA followed by Sidak’s post hoc test (95% CI) at *P < 0.05, **P < 0.01, ***P < 0.001. Dashed lines indicate the area of corpus callosum. (Scale bar: 100 µm.)

To analyze the observed changes in myelin integrity of CAG-Env mice in greater details, we analyzed a number of different oligodendroglial differentiation markers. In contrast to wt animals, transgenic CAG-Env mice displayed significantly reduced numbers of platelet-derived growth factor receptor-α (Pdgfrα)-positive oligodendroglial precursor cells (OPCs) in the course of CPZ treatment (Fig. 2 *A* and *B*). This is most likely resulting from fewer proliferating OPCs in the early phases of CPZ treatment (5 wk CPZ) as revealed by anti-Pdgfrα/Ki67 costaining (Fig. 2 *F* and *G*). To further analyze oligodendroglial differentiation, SRY-Box transcription factor 10 (Sox10) and adenomatous-polyposis-coli (APC) staining was performed. Sox10-positive/APC-negative cells correspond to differentiating OPCs and were found to be significantly reduced in numbers in CAG-Env mice compared to wt animals throughout demyelination and remyelination (Fig. 2 *A* and *C*). The number of Sox10/APC double-positive maturing oligodendrocytes was also found to be impaired in transgenic brains particularly upon CPZ withdrawal hence in the remyelination phase (Fig. 2 *A* and *D*). Furthermore, densities of early (re)myelinating oligodendrocytes marked by their expression of the breast carcinoma amplified sequence 1 (Bcas1) protein (27), were also significantly reduced in transgenic tissues at all stages analyzed. Of note, no differences in relation to myelin and oligodendroglial parameters were observed in control (untreated) wt and transgenic mice (Figs. 1 *E* and *F* and 2 *A*–*E*). These observations clearly demonstrate that transgenic expression of the HERV-W ENV protein indeed affects oligodendroglial differentiation in vivo at multiple levels leading to impaired myelination - thereby corroborating our previous ex vivo findings (17, 18).

### Impaired Demyelination and Remyelination Are Characterized by HERV-W ENV-Driven Activation of Microglial Cells.

**Fig. 3.**
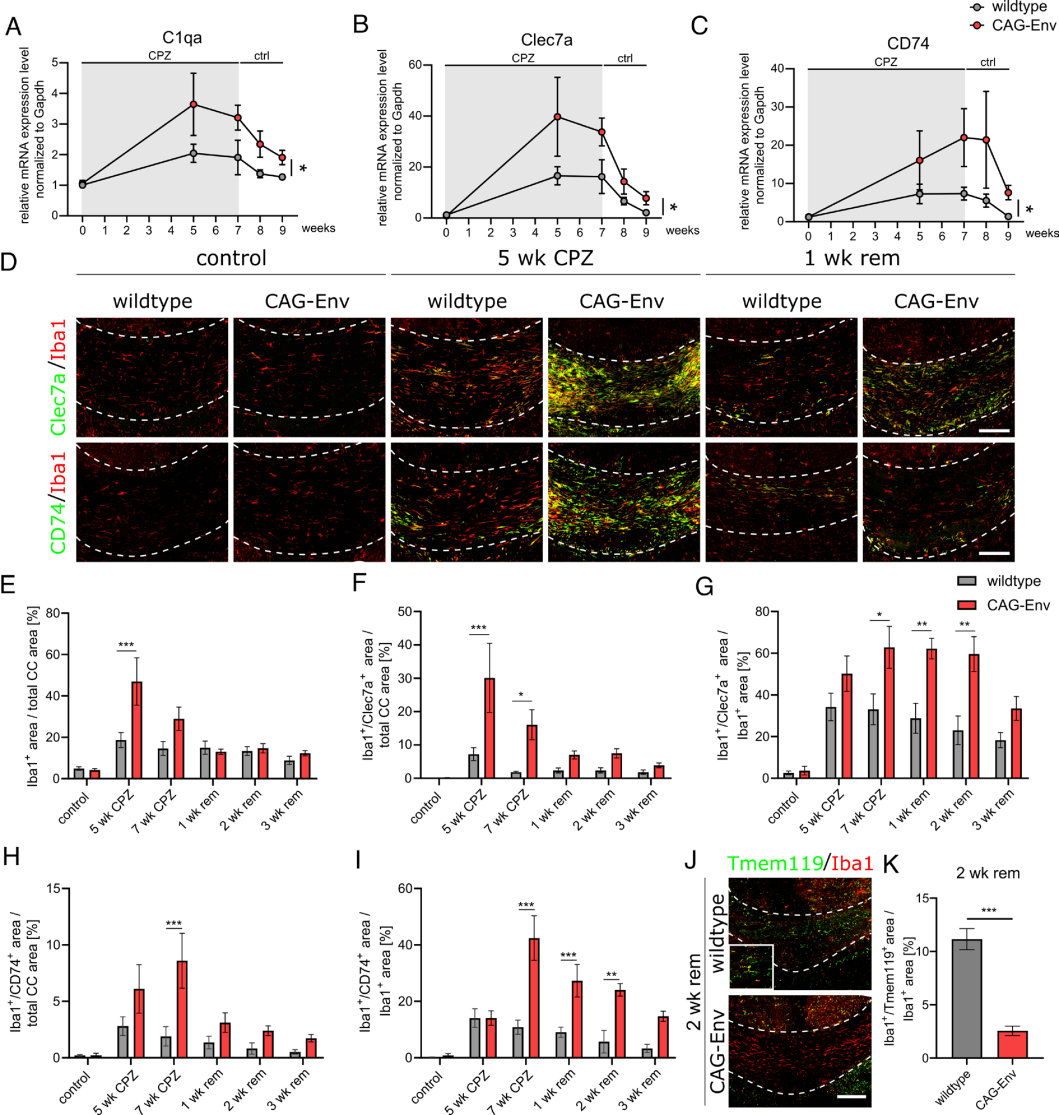
Transgenic HERV-W ENV expression activates microglial cells upon CPZ treatment. (*A*–*C*)C1qa, Clec7A, and CD74 gene expression analysis in the corpus callosum before and during CPZ treatment in wt vs. transgenic CAG-Env mice. (*D*) Representative immunohistochemical pictures of Clec7a/Iba1- and CD74/Iba1 coexpressing cells in wt and transgenic corpus callosum tissues (unchallenged and CPZ-treated animals). (*E*) Quantification of the Iba1-positive area over the total corpus callosum area in control vs. CPZ-treated wt and CAG-Env mice. (*F*) Quantification of Clec7a/Iba1 double-positive areas over total corpus callosum areas in wt and transgenic CAG-Env mice (control- and under CPZ diet). (*G*) Quantification of the proportion of Clec7a-positive microglia in wt and transgenic mice (control and under CPZ diet). (*H*) Quantification of CD74-positive microglial vs. total corpus callosum areas in wt and CAG-Env mice (control and under CPZ diet). (*I*) Quantification of the proportion of CD74-positive microglia in wt and transgenic mice (control- and under CPZ diet). (*J*) Representative immunohistochemical images of Tmem119/Iba1 coexpressing cells in wt and transgenic corpus callosum tissues at 2 wk of remyelination. (*K*) Quantification of the proportion of Tmem119-positive microglia in wt and transgenic mice at 2 wk of remyelination. Data are presented as mean values (n = 6) ± SEM. Significance of gene expression analysis was assessed by a Student’s unpaired *t* test of calculated AUCs whereas statistical significance of histological data was analyzed via 2-way ANOVA followed by Sidak’s post hoc test. Data were considered as statistically significant (95% CI) at *P < 0.05, **P < 0.01, ***P < 0.001. CC = corpus callosum. Dashed lines in (*D* and *J*) demarcate the corpus callosum. (Scale bar in *D* and *J*: 100 µm.)

We previously reported that the HERV-W ENV protein polarizes microglial cells ex vivo toward an axon damaging phenotype (11). Current advances in characterizing reactive microglia at molecular levels throughout a number of CNS diseases and models lead to the description of disease-associated microglial gene signatures and markers. A few of these disease-associated markers were used to study reactive microglia in the CAG-Env transgenic mouse model. C-type lectin domain family 7 member A (Clec7a) was described as highly up-regulated gene associated with different neurodegenerative diseases (28–30), whereas, complement C1q subcomponent subunit A (C1qa) was described as a key signaling protein of disease-associated microglia leading to the neurotoxic activation of astrocytes (31). Cluster of differentiation 74 (CD74) was found to be induced in activated microglia also associated with neurodegenerative diseases and aging (28, 32, 33). We performed gene expression analysis of extracted corpus callosum tissue and observed that all three genes (Clec7a, CD74, and C1qa) were expressed at similar levels in untreated mice but significantly induced during the course of CPZ induced demyelination and remyelination in transgenic CAG-Env mice as compared to wt animals [Fig. 3 *A*–*C*; as revealed by calculating area under the curve (AUC) values]. Immunohistochemical staining against the ionized calcium-binding adapter molecule 1 (Iba1), Clec7a and CD74 proteins was performed next (Fig. 3*D*). Analyzing the degree of Iba1-positive areas in the corpus callosum already indicated, that transgenic animals experienced a stronger microglial activation as compared to wt mice especially at earlier time-points (5 and 7 wk of CPZ treatment; Fig. 3 *D* and *E*). Unchallenged wt and CAG-Env mice displayed no differences in Iba1-positive areas as well as in Iba1/Clec7a or Iba1/CD74 double-positive areas (Fig. 3 *D*–*I*). At all stages of CPZ-treatment, Clec7a-positive microglial cells were significantly increased in transgenic mice (Fig. 3*D*) identified by increased Iba1/Clec7a double-positive areas (Fig. 3*F*). When normalized against total Iba1-positive areas (Fig. 3*G*), the proportion of Clec7a-positive, disease-associated microglia was significantly elevated upon transgene expression. While peaking slightly later, the analysis of Iba1/CD74 double-positive cells further confirmed the activated microglial phenotype in transgenic mice under CPZ application (Fig. 3 *D*, *H*, and *I*), which was followed by a reduced presence of homeostatic (Tmem119 expressing) microglial cells during the remyelination phase (Fig. 3 *J* and *K*), identified by decreased Tmem119/Iba1 double-positive areas.

### HERV-W ENV Protein-Driven Activation of Astrocytes.

Besides microglial activation, astroglial cell polarization is of further interest in the context of neurodegenerative diseases. Yet direct HERV-W ENV-dependent effects exerted on astroglia have not been reported, despite the fact that they express toll-like receptor 4 (TLR4) (34), one of the receptors for HERV-W ENV (7). However, several markers for activated and/or neurotoxic astrocytes including the complement cascade have recently been described as being induced in CNS pathologies. Complement component 3 (C3) and especially its cleaved form C3d was described in astrocytes from MS patients but also in different MS models (31, 35). In contrast to the general activation marker C3d, lipocalin-2 (Lcn2) was assigned as neurotoxic astrocyte marker, as the secreted protein can induce neuronal death (31, 36). In order to gain first insights into astroglial activation, C3 and Lcn2 gene expression analysis of isolated corpus callosum tissue was performed.

**Fig. 4.**
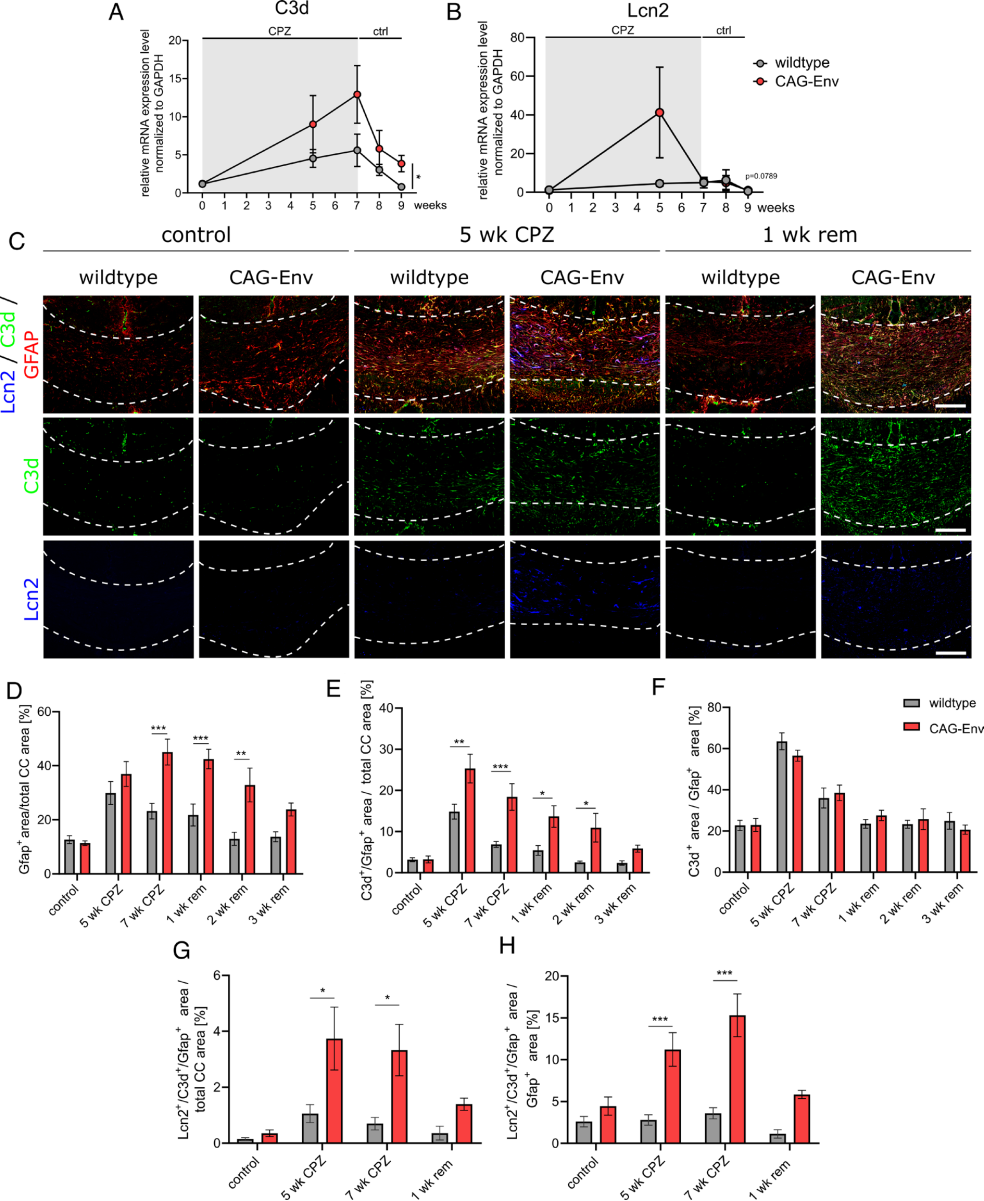
Transgenic expression of HERV-W ENV activates astrocytes. (*A* and *B*) C3d and Lcn2 expression gene expression analysis at various time points, before, during and after CPZ treatment in wt vs. transgenic CAG-Env mice. (*C*) Representative immunohistochemical images of C3d-, Lcn2- and Gfap- (co)expressing cells in wt and transgenic corpus callosum tissue sections (control and CPZ treated). (*D*) Quantification of Gfap-positive areas within the corpus callosum of wt and CAG-Env mice (control and CPZ treated). (*E*) Quantification of C3d/Gfap double-positive areas within the corpus callosum of wt and CAG-Env mice (control and CPZ treated). (*F*) Relative proportion of C3d/Gfap double-positive astrocytic areas in wt and CAG-Env animals (control- and CPZ treated). (*G*) Analysis of Lcn2/C3d/Gfap triple-positive astrocytic areas with the corpus callosum of wt and transgenic mice (control and CPZ treated). (*H*) Relative proportion of Lcn2/C3d/Gfap triple-positive astrocytic areas within the corpus callosum of transgenic and control mice upon CPZ treatment. Data are presented as mean values (n = 6) ± SEM. Significance of gene expression analysis was assessed by Student’s unpaired *t* test of calculated AUCs whereas the statistical significance of histological data was analyzed via 2-way ANOVA followed by Sidak’s post hoc test. Data were considered as statistically significant (95% CI) at *P < 0.05, **P < 0.01, ***P < 0.001. CC = corpus callosum. Dashed lines in *C* demarcate the area of the corpus callosum. (Scale bar in *C*: 100 µm.)

C3 expression was significantly increased in CAG-Env corpus callosum tissue upon CPZ treatment, whereas control (unchallenged) animals displayed no difference in transcript levels between wild-type (wt) and transgenic mice (Fig. 4*A*). Lcn2 displayed a different expression profile with a strong peak of expression in transgenic corpus callosum tissue after 5 wk of demyelination, whereas in wt litter mates only a mild increase in expression peaking at 2 wk post CPZ feeding was observed (Fig. 4*B*). Again, control animals showed no difference in Lcn2 expression. To corroborate this observation at protein level, glial fibrillary acidic protein (Gfap)-positive astrocytes were evaluated for their expression of C3d and Lcn2 proteins by immunohistochemistry (Fig. 4*C*). As Gfap itself is often already described as an activation marker for astrocytes in vivo, Gfap-positive areas were quantified upon CPZ treatment. This revealed that in transgenic mice astroglial activation was increased in both phases, under CPZ treatment and during remyelination, identified by increased GFAP-positive areas (Fig. 4*D*). Analyzing C3d/Gfap double-positive areas then demonstrated a significant elevation of C3d-positive astrocytes in the corpus callosum of transgenic animals vs. wt mice (Fig. 4*E*). However, the proportion of C3d-positive astrocytes normalized to all Gfap-positive astrocytes remained unchanged (Fig. 4*F*). Since Lcn2 expression was found to be particularly limited to the active demyelination phase (Fig. 4*B*), quantification of Gfap/C3d/Lcn2 triple-positive cells was performed and confirmed a strong induction of neurotoxic astrocytes during CPZ treatment (Fig. 4*G*). However, in contrast to C3d the proportion of Lcn2-positive astrocytes over all activated astrocytes changed with significantly elevated cell densities after 5 and 7 wk of CPZ treatment (Fig. 4*H*). Naïve (control) animals, again, showed no significant differences in the occurrence of Gfap, Gfap/C3d double-positive as well as Gfap/C3d/Lcn2 triple-positive cells (Fig. 4 *C*–*H*). These observations suggest that transgene expression generally increases astroglial activation levels and specifically boosts the presence of neurotoxic phenotypes.

### HERV-W Dependent Activation of Microglia and Astrocytes Ex Vivo.

**Fig. 5.**
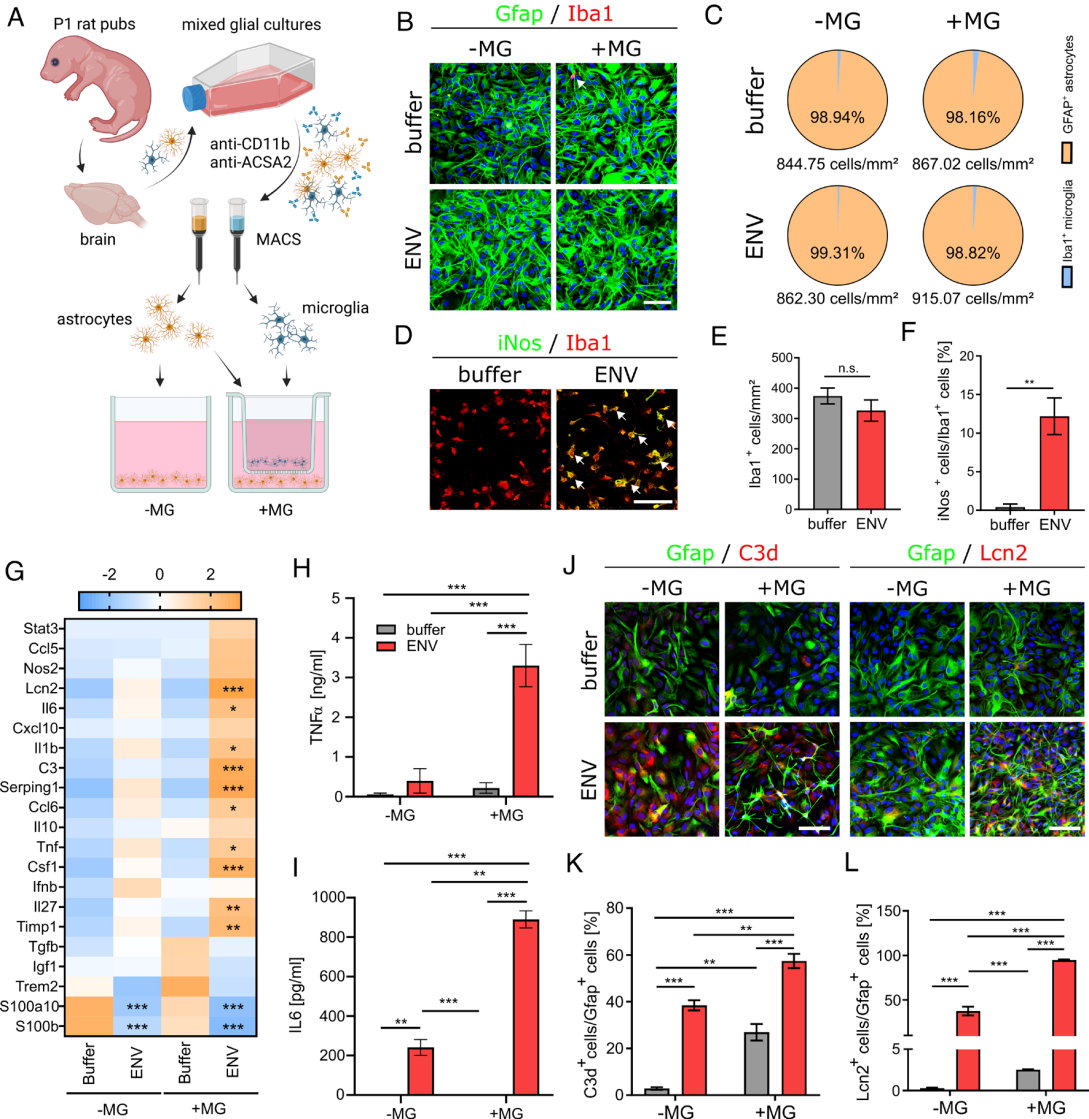
HERV-W ENV protein exposure leads to an activation of astroglial cells, which is amplified by microglia. (*A*) Schematic presentation of experimental procedure to generate spatially separated primary cultures of microglia and astrocytes (created using BioRender.com). (*B*) Representative immunocytochemical images of Gfap-positive astrocytes in absence and presence of microglia, treated with buffer or recombinant HERV-W ENV protein for 24 h. (*C*) Quantification of astrocyte culture purities under all four conditions. (*D*) Representative immunocytochemical images of microglial cells grown on cell culture inserts expressing Iba1 and iNos. Arrows point to iNos-positive cells. (*E* and *F*) Quantification of Iba1-positive and iNos-positive microglia densities upon buffer and ENV protein stimulation after 24 h. (*G*) Astrocyte gene expression analysis in absence and presence of microglia and in response to buffer or recombinant HERV-W ENV protein treatment after 24 h. Data are presented as z-scores. (*H* and *I*) Quantification of sandwich ELISA using media collected in absence and presence of microglia and in response to buffer or recombinant HERV-W ENV protein treatment after 24 h, identifying secreted TNFα (*H*) and IL-6 (*I*). (*J*) Representative immunocytochemical images of C3d and Lcn2 expressing (Gfap-positive astrocytes) under all four conditions and after 24 h. (*K* and *L*) Quantification of C3d-positive and Lcn2-positive astrocytes under all four conditions and after 24 h. Data are presented as mean values (n = 3) ± SEM. Significance of microglia analyses (*E* and *F*) was assessed by Student’s unpaired *t* test whereas the significance of qPCR quantifications (*G*) was assessed using 2-way ANOVA followed by false discovery rate post hoc test. All other quantifications (*H*, *I*, *K*, and *L*) were analyzed via 2-way ANOVA followed by Sidak’s post hoc test. Data were considered as statistically significant (95% CI) at *P < 0.05, **P < 0.01, ***P < 0.001. (Scale bar: 50 µm.) The arrow in *B* points to a single Iba1-positive microglial cell. Arrows in *D* point to iNOS-positive microglial cells.

It has previously been demonstrated that astrocytes can be activated via TLR4 receptor signaling (34) but also in response to microglial cytokines (31). In the HERV-W context, we already described that microglial cells express and secrete proinflammatory cytokines such as tumor necrosis factor-α (TNFα), interleukin (IL)-1β and IL-6 (11) as well as the above described C1qa (Fig. 3*A*). We therefore examined to what degree observed astrocyte reactions, were directly elicited by the ENV protein or mediated by ENV-activated microglia (11). To this end, we isolated microglial and astroglial cells from rat primary mixed glial cultures using magnetic-activated-cell-sorting (MACS) and combined these two cell types preventing direct cell contacts but enabling exchange of cytokines and signaling peptides (Fig. 5*A*). These cultures were then stimulated for 24 h with 1 µg/mL recombinant HERV-W ENV protein. Anti-Gfap, anti-Iba1 immunocytochemistry revealed >98% pure astrocytes and that no microglial cells were able to cross the barrier of the cell culture insert (Fig. 5 *B* and *C*). Since HERV-W ENV activated microglia displayed a strong induction of inducible nitric oxide synthases (iNOS) expression (11) cell culture inserts were stained using anti-Iba1 and anti-iNOS antibodies confirming microglial activation as well as stable cell densities (Fig. 5 *D*–*F*). To analyze the activation status of astroglial cells, the expression of state describing genes were quantified via qPCR (Fig. 5*G*). Interestingly, S100a10 and S100b expression were significantly down-regulated upon HERV-W ENV treatment regardless of whether microglia were present or not, whereas Serping1, C3 and Lcn2 transcript levels were induced. Although all these proinflammatory genes were already induced by the ENV protein alone, their expression was further boosted in the presence of ENV-reactive microglial cells. Additionally, expression levels of proinflammatory cytokines such as Tnf, Il1b, Il6 were also significantly induced. Validation at protein levels demonstrated that TNFα secretion (Fig. 5*H*) was primarily induced by ENV-challenged microglia, while IL-6 secretion was already significantly induced in astrocytes cultivated without microglia (Fig. 5*I*). These findings were additionally corroborated with Gfap-positive astrocytes expressing C3d and Lcn2 proteins in response to ENV exposure and being amplified in presence of microglial cells (Fig. 5 *K*–*M*).

### Transgenic HERV-W ENV Expression Leads to an Aggravated course of Experimental Autoimmune Encephalomyelitis.

**Fig. 6.**
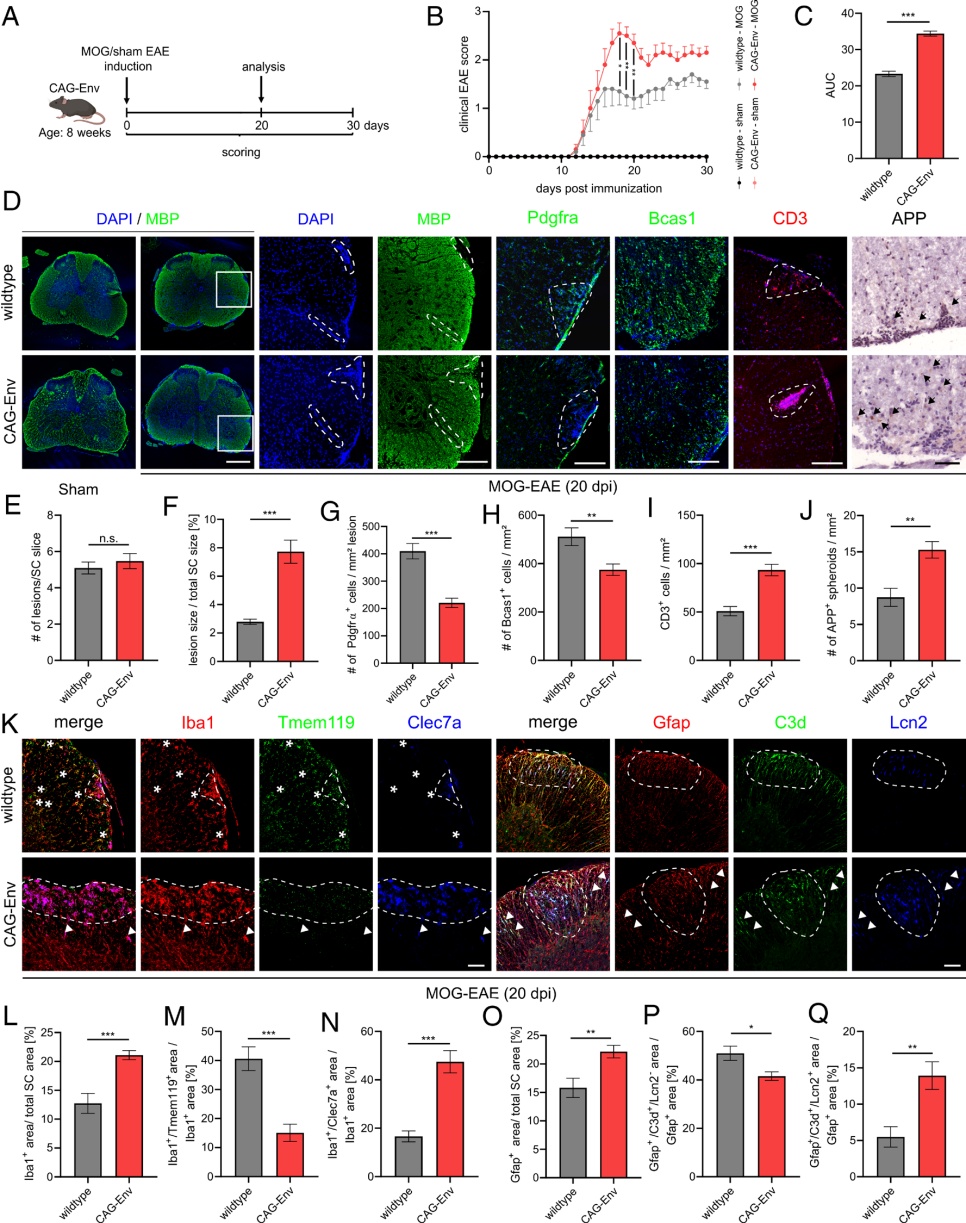
Increased microglial and astroglial activation in transgenic mice upon induction of EAE. (*A*) Schematic representation of EAE experiments. (*B*) Clinical score of sham- and MOG-induced wt and CAG-Env mice. (*C*) Determination of AUC values of clinical scores in MOG-induced wt vs. CAG-Env mice. (*D*) Representative images of anti-MBP, anti-Pdgfrα, anti-Bcas1, anti-CD3 and anti-APP-stained lumbar spinal cord sections of sham vs. MOG-induced wt and transgenic animals (20 dpi). (*E* and *F*) Quantification of lesion formation in MOG-induced animals displaying no differences in lesion numbers (*E*) but increased lesion sizes in CAG-Env mice (*F*) at 20 dpi. (*G*) Quantification of lesion-associated Pdgfrα-positive OPCs in wt and CAG-Env animals (20 dpi). (*H*) Analysis of Bcas1-positive myelinating oligodendrocytes in wt and transgenic animals (20 dpi). (*I*) Quantification of CD3-positive T cells in wt vs. CAG-Env mice (20 dpi). (*J*) Analysis of APP-positive spheroids in spinal cords of wt and transgenic animals (20 dpi). (*K*) Representative images of Iba1- and Clec7a expression patterns in lumbar spinal cords of MOG-induced wt vs. CAG-Env mice (20 dpi) as well as representative images of Gfap-, C3d-, and Lcn2 expression patterns in lumbar spinal cords of MOG-induced wt vs. transgenic animals (20 dpi). (*L*) Quantification of Iba1-positive areas (over total spinal cord areas) and (*M*) Tmem119-positive areas (within Iba1-positive areas) as well as (*N*) of Clec7a-positive areas (within Iba1-positive areas) in MOG-induced wt and CAG-Env mice at 20 dpi. (*O*) Quantification of Gfap-positive areas (over total spinal cord areas) in wt and CAG-Env mice at 20 dpi. (*P*) The proportion of C3d-positive but Lcn2-negative astrocytic areas relative to total Gfap-positive areas was reduced in transgenic CAG-Env mice. (*Q*) On the other hand, the extent of Lcn2/C3d/Gfap-triple-positive areas (within Gfap-positive areas) was significantly increased in transgenic animals at 20 dpi. Data are presented as mean values ± SEM. EAE course (*B*) was analyzed in n = 10 mice and histological analysis (*D*–*Q*) was performed in n = 9 animals. Significance of the clinical EAE score (*B*) was assessed via 2-way ANOVA followed by Sidak’s post hoc test and all other data were analyzed by Student’s unpaired *t* test. Data were considered as statistically significant (95% CI) at *P < 0.05, **P < 0.01, ***P < 0.001. [Scale bar in *D* (overview): 500 µm; scale bar in (*D*) (detailed): 200 µm; all other scale bars in *D* and *K*: 50 µm.] Dashed lines in (*D* and *K*) delineate lesion boundaries and arrowheads point to either Clec7a-positive microglia (*K*, *Left*) or Lcn2/C3d-positive astrocytes (*K*, *Right*) outside lesions.

In order to analyze the effects of transgenic HERV-W ENV expression in the context of neuroinflammation, we applied myelin oligodendrocyte glycoprotein fragment 33-55 peptide-induced experimental autoimmune encephalomyelitis (EAE, Fig. 6*A*). By analyzing the clinical score of diseased mice, a significant worsening of symptoms could be observed in CAG-Env mice as compared to wt litter mates particularly in the period between 18 to 20 days post induction (dpi) and thereafter (Fig. 6 *B* and *C*). Animals were then perfused at 20 dpi, and the lumbar spinal cords were analyzed in terms of lesion formation (anti-MBP immunohistochemistry, Fig. 6*D*). No differences regarding lesion numbers were observed between transgenic and wt mice (Fig. 6*E*), but relative lesion sizes were significantly increased in response to the transgene expression (Fig. 6*F*). In order to analyze OPC recruitment and oligodendroglial differentiation/myelination, numbers of lesion-associated OPCs (anti-Pdgfrα, Fig. 6 *D* and *G*) as well as of myelinating oligodendrocytes (anti-Bcas1, Fig. 6 *D* and *H*) were examined. This revealed a significant decrease in OPC recruitment and oligodendroglial differentiation in transgenic CAG-Env animals compared to wt litter mates (20 dpi). Since T-lymphocytes are thought to drive EAE development as well as to induce cytotoxicity in microglia (37), we analyzed the density of infiltrating T cells via CD3 immunohistochemistry (Fig. 6 *D* and *I*). The number of CD3-positive T cells was significantly increased in transgenic mice compared to wt animals. Furthermore, we performed anti-APP immunohistochemistry in order to confirm occurring neurodegeneration, revealing increased densities of APP-positive spheroids in spinal cords of CAG-Env mice compared to wt littermates upon EAE induction (20 dpi; Fig. 6 *D* and *J*).

Further immunohistological characterization of lesions then revealed that also Iba1-positive areas were significantly elevated in CAG-Env mice compared to wt animals (Fig. 6 *K* and *L*). However, assessing the proportion of Tmem119-positive homeostatic microglia revealed a significant reduction in transgenic CAG-Env mice compared to wt animals (Fig. 6 *K* and *M*). In parallel, when analyzing Clec7a-positivity a pronounced increase in areas harboring neurotoxic microglia and macrophages was observed (Fig. 6 *K* and *N*). Interestingly, in transgenic mice Clec7a-positive myeloid cells were also found outside lesion cores, indicating that neuroinflammation was less focally restricted in mutant mice (Fig. 6*K*, arrowheads). Accordingly, Tmem119-positive cells were also diminished outside of lesions in CAG-Env mice compared to nontransgenic litter mates (Fig. 6*G*, asterisks). Moreover, astroglial activation was examined via Gfap-, C3d- and Lcn2 expression (Fig. 6*G*). Quantification revealed a significant rise in relative Gfap-positive areas in the transgenic background (Fig. 6*O*). This was accompanied by increased areas featuring neurotoxic astrocytes (Gfap/C3d/Lcn2 triple-positive area; Fig. 6*P*), whereas areas with only activated astrocytes (Gfap/C3d-positive cells that lack Lcn2 expression) were slightly reduced (Fig. 6*Q*).

## Discussion

Activation and expression of this endogenous retroviral element has only been described in a few pathological instances such as in MS, chronic inflammatory demyelinating polyradiculoneuropathy, type-1 diabetes as well as in neurodevelopmental disorders (22, 38, 39). In addition, most recent evidence points to a strong activation in some Covid19 patients (40, 41). While initially discovered in MS patient-derived leptomeningeal cells (3), most information regarding impact and functions of HERV-W and its ENV protein relates to this autoimmune disease with immune, endothelial, oligodendroglial, and microglial cells being implicated (39, 42, 43). However, data on the functional role of this human-specific pathogenic entity have so far only been gathered from histopathological observations on autopsy material combined with ex vivo primary cell- and tissue-based functional assays. Yet, a direct proof of concept on the cellular responses evoked by the expression of the HERV-W ENV protein in vivo was missing so far.

We here describe strong reactions of the three glial cell types in response to transgenic HERV-W ENV expression, most notably only under MS related pathological conditions (EAE and CPZ), with microglial and astroglial phenotypes in accordance with recent descriptions of disease-associated glial signatures (30, 31, 44, 45). It is the first account of the generation of an overall neurotoxic environment, of worsening pathologies and of impeded regeneration processes, confirming and expanding previous postulations (5, 11, 18). In this study, we focused on the roles of glial cells since our earlier investigations pointed to an inhibited OPC differentiation as well as to neurotoxic microglial polarization. Moreover, glial pathological reactions were also suggested by the outcome of clinical trials with a neutralizing anti-HERV-W ENV antibody (temelimab) where reduced brain atrophy rates (suggesting a role in smoldering neurodegeneration) as well as preserved myelin integrities (supporting evidence for effects on oligodendroglial differentiation and myelin repair) were observed (19). Such an emphasis on degeneration and regeneration was additionally justified as these pathological processes still represent unmet clinical needs.

Astrocyte activation was unexpected and thus represents an interesting observation. These cells appear to be sensitive to both, a direct impact of the ENV protein as well as to signals emanating from polarized microglial cells – pointing to cell/cell interactions as recently described in chronic MS (46). This finding certainly contributes to the understanding of the emerging pathological role of astrocytic cells in MS and related demyelinating diseases (47), promoting research into cell-specific modularity approaches aiming at therapeutic opportunities. It will also be of interest to see whether and at what disease stages the here-described glial phenotypes can also be seen in MS tissue samples.

Our view on this viral protein’s impact on myelin repair has also been refined. ENV appears not only to interfere with oligodendroglial maturation, but its presence also resulted in a lack of OPC recruitment mostly due to a reduction in proliferation rates. This was not seen in former ex vivo studies due to the use of postmitotic primary cells. At present it is not known whether this relates to a direct effect on OPCs or whether lack of trophic input or the presence of an inhibitory milieu generated by microglia and/or astrocytes account for decreased cell numbers (48) -an issue that remains to be addressed in future studies.

Astrocyte and microglia/macrophage numbers were, however, increased in both lesion models also correlating with the larger lesions found in inflamed spinal cords of the EAE mice. Of note, in the transgenic background the degree of neurotoxic glial cells exceeded this ratio, arguing for a pronounced activation of neurotoxic phenotypes. Likewise, homeostatic cell numbers were reduced in both models. Given that in mutant mice reactive glial subtypes were also detected outside EAE lesions, a possible contribution to lesion growth can be suggested.

Finally, increased axonal degeneration as seen by the development of APP-positive spheroids was observed in transgenic tissues of both CPZ demyelinated, as well as EAE-challenged mice. It is thus tempting to speculate that observed aggravated clinical symptoms in the EAE model result (in part) from an enhanced degeneration processes. This on the one hand confirms our previous description of an axon-degenerating microglial phenotype in response to ENV protein exposure (11), on the other hand, it clearly demonstrates that even in a rather gentle and regenerative lesion set-up such as mediated via CPZ feeding, mutant animals are more impaired—a remarkable consideration for this model system (49).

While we could prove that all CNS glial cells respond to this pathological protein expression in two different models mimicking MS features, additional observations in the EAE model provided evidence that also autoimmune processes might be altered, given that lesions were not more frequent but larger in size and were thus containing more Iba1-positive (myeloid) cells. It is unlikely that this can be explained via microglia phenotype consolidation only, also in light of the fact of a more pronounced macrophage as well as T cell contribution known to occur in EAE. While we were able to identify an increased infiltration of CD3-positive T cells in EAE-challenged transgenic mice, it remains to be shown whether previous reports on Th1-like differentiation or superantigen-like activation of T cells (6, 7) can be confirmed in the in vivo model. Such a more detailed description of immune cells is out of scope of this study, and this also includes analyses of stem cells niches, endothelia as well as pericytes, all of which also being functionally implicated in autoimmune and neurodegenerative pathologies. Of note, a direct effect of HERV-W ENV exerted onto lymphoid cells was not supported by the clinical trials (19). But, it must be kept in mind that this circumstance might relate to the particular MS patient cohort enrolled for these trials as for example addressing early autoimmunity events, such as for example conversion from clinically isolated syndrome to relapsing-remitting MS, was not possible.

A notable limitation of our investigation relates to the applied transgenic mouse model. As we used a general and noninducible transgene expression, possible role(s) in development can currently not be excluded. Yet, using multiple protein markers related to oligodendrogenesis, microglial- and astroglial phenotype generation, we detected no differences between unchallenged (non-CPZ, non-EAE) transgenic mice and wt litter mates – at least at all ages analyzed here (Figs. 1–4 and 6). Also, no evidence of increased or predetermined axon degeneration was found providing further evidence that the model is valid and that HERV-W ENV obviously needs a pathological environment (toxin, infection, autoimmunity) to manifest its functionality as discussed previously (38, 39). Given the still rather unclear HERV-W activation process(es) involved in the pathology of MS, one might indeed suggest that this entity is part of a two-hit model the sequence of events still remains to be determined (38). Moreover, more accurate overexpression models await the still nonexisting information on the genomic nature of this pathological HERV-W element as it is currently not known to what degree fixed or unfixed copies and under which regulatory sequences account for activation and expression in the diverse diseases. Likewise, it will be difficult to measure any cognitive impairments or changes at subneuronal levels (synapses, plasticity) using the here-applied short-term experimental paradigms. Given the recent description of HERV-W ENV-modulating synapse maturation in vitro (50) but also taking into account findings on the neutralizing antibody temelimab conferring a rescue from brain volume loss (19), such important aspects need to be addressed in more chronic demyelination/degeneration scenarios.

Moreover, given the identification of EBV as being involved in the generation of MS on the long term (15), a more recent description of antibodies directed against EBV- and HERV-W-related proteins in the CSF of MS patients (51) further supports exogenous to endogenous viral entities to be implicated in different disease processes. Such an activation scenario has previously already been suggested (13, 14). Besides EBV, also other viral entities such as herpes simplex virus-1 or human herpesvirus-6 have been implicated in the pathogenesis of MS as well as in the activation process of HERVs (39). Interestingly, these viruses were also shown to be able to infect neural and glial cells (52–54).

In conclusion, we here present the long expected functional proof of HERV-W ENV’s degenerative potential in vivo and demonstrate that mainly glial cells react and contribute to the generation of a neurotoxic parenchyma. It is tempting to speculate that our findings not only relate to pathological processes underlying MS but also to tissue changes in neurodevelopmental disorders or in long-Covid19 patients. Therefore, the further development of suitable neutralization strategies such as by preclinical and clinical examinations of the temelimab antibody or of unrelated pharmacological approaches (17, 55) is highly warranted.

## Materials and Methods

### Ethics Statements for Animal Experiments.

Animal experiments were performed following the ARRIVE guidelines and according to the NIH guide for the care and use of Laboratory animals (NIH Publications No. 8023, revised 1978). The Institutional Review Board of the ZETT (Zentrale Einrichtung für Tierforschung und wissenschaftliche Tierschutzaufgaben) at the HHU (Heinrich-Heine-University Düsseldorf) permitted tissue isolation procedures (O69/11, O90/15). The review board of the state government Landesamt für Natur, Umwelt und Verbraucherschutz Nordrhein-Westfalen, North-Rhine Westphalia, Germany approved all animal experimental procedures under licenses: Az.:84-02.04.2017.A137 and 81-02.04.2019.A063.

### Animal Models.

Transgenic C57BL6/J;129P2/Ola-Hprttm(CAG-Env) (CAG-Env; 75% C57BL6/J + 25% 129P2/Ola) mice were used in this study. Animal genotypes were determined using genomic PCR for hypoxanthine-guanine phosphoribosyltransferase (Hprt) alleles as established previously (22). Briefly, genomic DNA was extracted from ear punches used for animal labelling according to the manufacturers protocol (PureLink genomic DNA-Minikit, Thermo Fisher Scientific, Waltham, MA, USA). Afterward, alleles were amplified using Red HS Taq Master Mix (Biozym, Hessisch Oldendorf, Germany) and analyzed on a 2% agarose gel (LE Agarose, Biozym). For the detection of Hprt wt and transgenic (tg) alleles following primer pairs were used: wt_fwd: 5′- TGT CCT TAG AAA ACA CAT ATC CAG GGT TTA GG; wt_rev: 5′- CTG GCT TAA AGA CAA CAT CTG GGA GAA AAA and tg_fwd: 5′-ACG TCA GTA GTC ATA GGA ACT GCG GTC G; tg_rev: 5′-TAC AGG CGT GAA CCA CTG CTC CCT using temperature cycles: 94 °C for 2 min, followed by 94 °C for 30 s, 55 °C (wt)/65 °C(tg) for 30 s, 68 °C for 60 s, repeated 35 times, then 68 °C for 8 min resulting in a wt DNA fragment of 345 bp and a transgenic (tg) DNA fragment of 399 bp. Mice were bred by the ZETT and housed in a pathogen-free facility (SPF) with 12 h light/dark cycle and supplied with food/water ad libitum. Demyelination was induced in 8-wk-old mice using a diet containing 0.2% w/w CPZ [bis(cyclohexanone)oxaldihydrazone] (V-1534, Ssniff, Soest, Germany) similar as previously described (24–26). In order to achieve sufficient demyelination, animals had to be fed for 7 wk with CPZ (Fig. 1 *E* and *F*) and were changed afterward to control diet without CPZ (V-1534, Ssniff) for 1, 2, and 3 wk (1-, 2-, 3-wk rem). Control animals (unchallenged, no lesion formation) received chow without CPZ (V-1534, Sniff). The diet was changed twice per week and animal bodyweights were monitored twice per week. All CPZ experiments were performed with 6 animals (either sex) per group and time point according to the cohort size analysis (using G*Power 3.1.9.7; effect size: 2.6; α-level: 0.05; Power: 0.95). EAE was induced as previously described (56). Briefly, 8-wk-old mice (either sex) were immunized with 200 µg myelin oligodendrocyte glycoprotein fragment 35-55 (MOG35-55) (Biotrend, Cologne, Germany) followed by intraperitoneal injections of 200 ng pertussis toxin (Sigma-Aldrich, St. Louis, MO, USA) at days 0 and 2. Afterward, animals were monitored and the clinical EAE score (0 to 5) (57) was determined on a daily basis for the following 30 d. Cohort size analysis for EAE experiments (using G*Power 3.1.9.7; effect size: 1.6; α-level: 0.05; Power: 0.95) resulted in an optimal group size of 12 animals. Furthermore, only animals that developed clinical signs of paralysis (EAE score) were included in subsequent analyses.

### Primary Rat Mixed Glial Cultures.

Primary rat mixed glial cultures containing microglia and astrocytes were isolated as previously described (11). Following the 10-d cultivation period, microglia were collected from flask supernatants that were shaken at 180 rpm/min at 37 °C. Subsequently, astrocytes and microglia were purified using MACS according to the manufacturer’s protocol (Miltenyi Biotec, Bergisch-Gladbach, Germany). Briefly, the remaining astrocytes were dislodged by trypsin-ethylenediaminetetraacetic acid-treatment (Thermo Fisher Scientific), labeled using a combination of anti-ACSA1-biotinylated antibodies in combination with antibiotin microbeads (Miltenyi Biotec) and applied to the isolation column. Microglial cells were detached by accutase treatment and labeled with anti-rat CD11b/c microbeads (Miltenyi Biotec). Cells were assessed for viability and counted under trypan blue staining. Cell purities as determined via Gfap/Iba1-staining were consistently >98%. Astrocytes (30,000 cells/well, 2 wells/ condition) and microglia (100,000 cells/transwell, 2 wells/condition) were seeded on 24-well plates and respective transwells in DMEM medium containing 10% FCS, 2 mM L-glutamine and 50 U/mL penicillin/streptomycin (Thermo Fisher Scientific). After 24 h, cell cultures were stimulated with either 1 µg/mL recombinant HERV-W ENV protein (Protein’eXpert, Grenoble, France) or reconstitution buffer. To avoid side effects through the recombinant production of HERV-W ENV protein, Endotoxin levels were measured using the limulus amebocyte lysate-test and found to be below the detection limit (<5EU = mL).

### Tissue Isolation for Transcript and Protein Expression analysis.

Briefly, animals were deeply anesthetized with isoflurane and transcardially perfused with 20 mL cold phosphate-buffered saline (PBS) to remove blood cells from the brain tissue. For the detection of HERV-W ENV protein using automated western blot techniques, whole brains were isolated and immediately frozen in liquid nitrogen. For CPZ experiments, the brain was isolated and placed in a murine brain matrix (BSMAS001-1; Zivic instruments, Pittsburg, USA) and three 1-mm corpus callosum (corpus callosum) containing slices were isolated and placed in a drop PBS. Immediately, corpus callosum was isolated using a binocular and scalpel and snap frozen in liquid nitrogen. All samples were stored at −80 °C until Protein and/or RNA isolation.

### RNA Extraction, cDNA Synthesis, and RT-qPCR.

For the RNA extraction from cell cultures, cells were lysed using 350 µL β-mercaptoethanol (Sigma-Aldrich)—RLT buffer (1:100, Qiagen, Hilden, Germany) and immediately snap frozen on dry ice. Afterward, total RNA was isolated using the column-based RNeasy mini kit (Qiagen) according to the manufacturer’s protocol. For the RNA extraction from snap frozen tissue, snap frozen tissue was homogenized by applying 1 mL TRIzol™ reagent (Thermo Fisher Scientific)/ 100 mg tissue using Polytron PT 2100 homogenizer (Kinematica AG, Malters, Switzerland). Afterward, total RNA was isolated according to the manufacturer’s protocol. RNA quality and concentration were quantified by a Nanodrop spectrophotometer (Thermo Fisher Scientific), and samples were stored at −80°C until analysis. Total RNA was reverse transcribed via the high-capacity cDNA Reverse Transcription Kit (Thermo Fisher Scientific). Determination of transcript levels was performed in a quantitative manner using a 7900HT sequence detection system (Thermo Fisher Scientific) and Power SybrGreen PCR master mix (Thermo Fisher Scientific) as previously published (11). The here used primer sequences (as determined by PrimerExpress 2.0; Thermo Fisher Scientific) are listed in *SI Appendix*, Table S1. Compared to Hprt, Odc, Tbp, Gapdh proved to be the most accurate and stable normalization gene and was therefore used as reference gene. Each sample was measured in duplicates and relative transcript levels were calculated according to the ΔΔCt method.

### Enzyme-Linked Immunosorbent Assay (ELISA).

To assess glial secretion of TNFα and IL-6, the following colorimetric sandwich ELISA kits were used: rat TNF alpha ELISA Kit (ab100785, Abcam) and rat IL-6 ELISA kit (ab234570, Abcam). Culture media were collected, spun down (1,000 × g; 5 min; 4 °C), and stored at −80 °C. Prior to use, all reagents were thawed and adjusted to room temperature. Culture media were measured in duplets according to the supplier’s protocol.

### Protein Isolation and Automated Western Blot analysis.

For the detection of HERV-W ENV antigen, snap frozen mouse tissue was extracted according to MEM-PER manufacturer’s instructions (Thermo Fisher Scientific). Mouse brains were homogenized with 3 cycles of 20 s of Precellys (CK14, Bertin instruments, Montigny-le-Bretonneux, France). After 10 min of centrifugation at 10,000 × g, supernatants were collected. HERV-W ENV antigen detection was analyzed on the Jess device using Simple Western technology an automated capillary-based size sorting and immunolabeling system (ProteinSimpleTM, Biotechne Miniapolis, MA, USA) as previously described (23). Anti-HERV-W ENV mAb GN_mAb_ENV01 (Geneuro, Geneva, Switzerland) was used at 20 µg/mL to detect antigen. HERV-W ENV antigen was identified within the apparent molecular weight range (350 to 450 KDa) in this capillary matrix, using the Jess platform Compass TM software (ProteinSimpleTM, Biotechne Miniapolis, MA, USA).

### Immunocytochemistry.

For immunocytochemistry, astroglial-microglial cocultures were fixed for 10 min with 4% paraformaldehyde (PFA), PBS washed, blocked for 45 min according to the host of the secondary antibody [either 2% normal goat serum (NGS) or 10% normal donkey serum (NDS) respectively, both Sigma-Aldrich] and 0.5% Triton X-100 (Sigma-Aldrich) in PBS. Afterward, cells were incubated at 4 °C overnight with primary antibody solution (as listed in *SI Appendix*, Table S2) containing, 10% NDS and 0,1% Triton X-100 in PBS. Following PBS washes species-appropriate Alexa fluorochrome-conjugated secondary antibody (1/200 in PBS, Thermo Fisher Scientific) and DAPI (20 ng/mL, Roche, Basel, Switzerland) were incubated for 30 min at RT. Afterward, coverslips were washed in PBS and embedded using Shandon™ Immu-Mount (Thermo Fisher Scientific).

### Immunohistochemical Procedures.

EAE- and CPZ-treated animals were deeply anesthetized with isoflurane and transcardially perfused with 20 mL cold PBS followed by 20 mL 4% PFA (Sigma-Aldrich) Brains and/or spinal cord were isolated and post-fixed in the same fixative for 1 d at 4 °C, followed by 24 to 48 h cryoprotective dehydration in 30% sucrose at 4 °C. Afterward, tissue was embedded in Tissue-Tek (Sakura Finetek Europe, Alphen aan den Rijn, Netherland), frozen and stored at −30 °C until preparation of 12-µm sections using a cryostat (Leica CM30510S, Leica, Wetzlar, Germany). For CPZ experiments, coronal sections of the caudal corpus callosum (Bregma: −0.70 to −2.06) were collected and for EAE experiments, transverse lumbar spinal cord sections were prepared. All sections were stored at −30 °C until immunohistochemical analysis avoiding any freeze–thaw cycles. To assess the relative myelination of the corpus callosum, LFB (Sigma-Aldrich) staining was used. Slides containing 12-µm coronal section were incubated overnight in LFB solution (0.1% LFB, 4% glacial acetic acid in 96% ethanol) at 56 °C. Afterward, redundant LFB staining was washed out using 0.05% lithium carbonate solution (in ddH2O), tissue was dehydrated and embedded in ROTI-Histokit II (Carl Roth, Karlsruhe, Germany). For immunohistochemistry, brain sections (CPZ) and/or spinal cord sections (EAE) were thawed and left to dry for 15 min at RT. Afterward, sections were rehydrated for 5 min in distilled water, post fixated for 5 min in 4% PFA and for another 5 min in −20°C acetone. Afterward, sections were washed once in Tris-buffered saline (TBS, pH 7.6), once in TBS-T (TBS containing 0.02% Triton) for 5 min each and incubated for another 5 min in 0.3% H2O2 solution. Blocking was performed using 10% NGS (Sigma-Aldrich) and 5% biotin-free bovine serum albumin (BSA; Sigma-Aldrich; in TBS-T) for 30 min at RT, followed by the application of the primary antibodies (*SI Appendix*, Table S2) in 10% NGS and 5% BSA in TBS over night at 4 °C. Afterward, sections were washed twice in TBS (5 min each) and a biotinylated secondary antibody [goat anti-rabbit (1/200; Vector Laboratories, Burlingame, CA, USA)] was added for 30 min. Next, sections were washed twice in TBS and ABC reagent was incubated for another 30 min according to the manufacturer’s protocol (Vectastain Elite ABC HRP kit; Vector Laboratories). Afterward, sections were washed again twice for 5 min in TBS and peroxidase substrate was added for 5 min at RT (ImmPact DAB; Vector Laboratories). The reaction was stopped by two washing steps in ddH2O, followed by hematoxylin nuclear stain (Carl Roth), dehydration, and embedding in ROTI-Histokitt II (Carl Roth). For Immunofluorescence staining, brain (CPZ) and/or spinal cord sections (EAE) were thawed, rehydrated for 5 min in distilled water, post fixated for 5 min in 4% PFA and for another 5 min in −20°C acetone. Before blocking, sections were washed once using TBS (TBS, pH 7.6) and once in TBS-T (TBS containing 0.02% Triton) for 5 min each. Blocking was performed with 10% serum according to the host of the secondary antibody (NGS or NDS respectively; Sigma-Aldrich) and 5% biotin-free BSA (Sigma-Aldrich; in TBS-T) for 30 min at RT, followed by application of the primary antibodies (in 10% NGS/NDS, 5% BSA, TBS; *SI Appendix*, Table S2) and incubation overnight. Sections were washed two times for 5 min in TBS and incubated with the species-appropriate Alexa fluorochrome-conjugated secondary antibody (1/200 in TBS, Thermo Fisher Scientific) and DAPI (20 ng/mL, Roche) for 30 min at RT. Afterward, sections were washed once in TBS and once in TBS for 5 min each and embedded using Shandon™ Immu-Mount (Thermo Fisher Scientific).

### Image Acquisition and Analysis.

Images of astroglial cell cultures as well as of LFB- and DAB-stained tissue sections were captured on an Axioplan 2 microscope (Zeiss, Jena, Germany). All other Images were performed at a Zeiss CLSM microscope 510 (CLSM 510, Zeiss) always using the same exposure times, laser intensities and digital gains. The quantification of all microscopic images was performed using ImageJ software (NIH, Bethesda, MD, USA). For the analysis of microglial-astroglial cocultures, 7 images per coverslip/insert and 2 coverslips/inserts per treatment were quantified, for the analysis brain/spinal cord tissue, for each marker setup 3 (CPZ) or 4 (EAE) sections were analyzed per condition and replicate. Scale bars were always adjusted to the respective microscope and all other setting (including thresholds) were identically applied to all images of a marker set. The in vitro analysis of iNOS-positive microglia as well as the number of C3/Lcn2-positive astrocytes were quantified manually, using the ImageJ tool “cell-counter”. Similar is true for the quantification of oligodendroglial differentiation marker Pdgfrα, Sox10, APC, Bcas1 and Ki67, as well as for the APP-positive spheroids. To analyze the relative myelination of the corpus callosum, images of LFB staining were transformed to gray scale, and the same threshold was applied to all images in order to creating a binary image. Afterward the area of the corpus callosum as well as the LFB-positive area was determined and relative LFB-positive myelinated areas were calculated.

For the analysis of double and/or triple immune-positive cells, merged images were uploaded in ImageJ software, channels were split and a median filter (3) as well as background reduction (50, sliding parabolic, except for Gfap+ cells) was applied to all channels. Afterward binary images were created, always using the same threshold for each channel of a marker setup, and the images were re-merged. Now a RGB color threshold was applied to detect and measure double and/or triple colocalizing areas, accordingly.

### Statistical Analysis.

Data are presented as mean values ± SEM in which n represents the number of independent replicates. Statistical analyses were conducted using Graph-Pad Prism 8.4.3 (GraphPad Software, San Diego, California USA). All data showed a normal distribution assessed by the Shapiro–Wilk test. Therefore, pairwise comparisons were analyzed using a two-tailed unpaired Student’s *t* test, whereas multiple comparisons were assessed by 2-way ANOVA followed by false discovery rate- or Sidak’s post hoc test. Furthermore, statistical significance of in vivo qPCR data was assessed using a two-tailed unpaired Student’s *t* test of the calculated AUC. The experimental groups were considered significantly different at *P < 0.05, **P < 0.01, ***P < 0.001.

## Supplementary Material

Appendix 01 (PDF)

## Data Availability

All study data are included in the article and/or *SI Appendix*.
