# Supplementary material for: Transgenic expression of the HERV-W envelope protein leads to polarized glial cell populations and a neurodegenerative environment
Source: Proc Natl Acad Sci U S A. 2023 Sep 11;120(38):e2308187120. doi: 10.1073/pnas.2308187120 (PMC10515160; doi:10.1073/pnas.2308187120)
Supplement: Supplementary file 1 — Appendix 01 (PDF) [file pnas.2308187120.sapp.pdf]

PNAS

Supporting Information for
Transgenic expression of the HERV-W envelope protein leads to polarized glial cell populations and a neurodegenerative environment

Joel Gruchot[1], Isabel Lewen[1], Michael Dietrich[1], Laura Reiche[1], Mustafa Sindi[1], Christina Hecker[1], Felisa Herrero[2], Benjamin Charvet[3], Ulrike Weber-Stadlbauer[2,4], Hans-Peter Hartung[1,5,6], Philipp Albrecht[1], Hervé Perron[3], Urs Meyer[2,4] and Patrick Küry[1,7]*

[1] Department of Neurology, Medical Faculty, Heinrich-Heine-University Düsseldorf, 40225 Düsseldorf, Germany.
[2] Institute of Pharmacology and Toxicology, University of Zürich-Vetsuisse, CH-8057 Zürich, Switzerland.
[3] GeNeuro Innovation, 69008 Lyon, France.
[4] Neuroscience Center Zurich, University of Zürich and ETH Zürich, CH-8057 Zürich, Switzerland.
[5] Brain and Mind Center, University of Sydney, Sydney, NSW 2050, Australia.
[6] Department of Neurology, Palacky University Olomouc, 77146 Olomouc, Czech Republic.
[7] Department of Neurology, University of Bern, CH-3010 Bern, Switzerland

* Correspondence to Prof. Dr. Patrick Küry
Email: kuery@uni-duesseldorf.de

This PDF file includes:

Tables S1 to S2

Table S1. qPCR primer list

Name	Forward sequence	Reverse sequence
HERV-Wenv	TTT ACT CCT CTT TGG ACC CT	ATC TGG GGT TCC ATT TGA AG
mC1qA	GCC TGT GTG CTG ACC ATG AC	GGGTGC TCG GCA GAC ATC T
mC3	CCG TGA ACA GGA GGA ACT TAA GG	ATG CTG CAG AAG GCT GGA TT
mCd74	CCA ACG CGA CCT CAT CTC TAA	AGG GCG GTT GCC CAG TA
mClec7a	CCT TGG AGG CCC ATT GC	GCA ACC ACT ACT ACC ACA AAG CA
mGapdh	AGG TTG TCT CCT GCG ACT TCA	CCA GGA AAT GAG CTT GAC AAA G
mLcn2	CCC TGT ATG GAA GAA CCA AGG A	GCA AAG CGG GTG AAA CGT T
rC3	GGT CTG CGG AAG TGT TGT GA	GGC GCT GGC AGC TGT ACT
rCcl5	CCA ACC TTG CAG TCG TCT TTG	TCT GGG TTG GCA CAC ACT TG
rCcl6	CAA TTA CCT GGG ATT CCC GG	TGG GAA TGC CTC ATT TGC AT
rCsf1	CGA GGT GTC GGA GCA CTG TA	TCA ACT GCT GCA AAA TCT GTA GGT
rCxcl10	GGG ATC CCT CTC GCA AGA A	CTC AGC GTC TGT TCA TGG AAG T
rGapdh	GAA CGG GAA GCT CAC TGG C	GCA TGT CAG ATC CAC AAC GG
rIfnb1	TGG AAG GCT CAA CCT CAG CTA	GGG TGC ATC ACC TCC ATA GG
rIgf1	AGA CGG GCA TTG TGG ATG A	ACA TCT CCA GCC TCC TCA GAT C
rIl10	CCC AGA AAT CAA GGA GCA TTT G	CAG CTG TAT CCA GAG GGT CTT CA
rIl1b	GAA ACA GCA ATG GTC GGG AC	AAG ACA CGG GTT CCA TGG TG
rIl27	GCT GCG CAG GGA ATT CAC	CCC TGA ACC TCA GAG AGC AGT T
rIl6	GTT GTG CAA TGG CAA TTC TGA	TCT GAC AGT GCA TCA TCG CTG
rLcn2	GGG CAG GTG GTT CGT TGT C	AGC GGC TTT GTC TTT CTT TCT G
rNos2	CTC AGC ACA GAG GGC TCA AAG	TGC ACC CAA ACA CCA AGG T
rS100a10	GCC ATC CCA AAT GGA GCA T	CCC CTG CAA ACC TGT GAA AT
rS100b	GAG CAG GAA GTG GTG GAC AAA	CAC TCC CCA TCC CCA TCT T
rSerping1	GAC AGC CTG CCC TCT GAC A	GCA CTC AAG TAG ACG GCA TTG A
rStat3	CCG GCC CTT AGT CAT CAA GA	TTG ACC AGC AAC CTG ACT TTT G
rTgfb	AAA CGG AAG CGC ATC GAA	TGG CGA GCC TTA GTT TGG A
rTimp1	CGC AGC GAG GAG TTT CTC AT	GGC AGT GAT GTG CAA ATT TCC
rTnf	AGC CCT GGT ATG AGC CCA TGT A	CCG GAC TCC GTG ATG TCT AAG T
rTrem2	CCA AGG AGC CAA TCA GGA AA	GGC CAG GAG GAG AAG AAT GG

Table S2. Antibody list

Target	Host	Dilution	Company	RRID
Iba1	rabbit	1:500	WAKO Pure Chemical Corporation, Osaka, Japan	RRID: AB_839504
iNos	goat	1:250	Abcam, Cambridge, UK	RRID: AB_301857
Gfap	chicken	1:1000	Abcam, Cambridge, UK	RRID:AB_304558
hC3d	rabbit	1:300	Agilent, Santa Clara, CA, USA	RRID:AB_578478
Lcn2	goat	1:100	R and D Systems, Minneapolis, MN, USA	RRID:AB_355022
APP	rabbit	1:200	Thermo Fisher Scientific, Waltham, MA, USA	RRID:AB_2533902
Pdgfrα	goat	1:250	R and D Systems, Minneapolis, MN, USA	RRID:AB_2236897
Ki67	rabbit	1:250	Abcam, Cambridge, UK	RRID:AB_302459
Sox10	rabbit	1:100	DCS Immunoline, Hamburg, Germany	RRID: AB_2313583
APC (CC1)	mouse	1:300	Sigma-Aldrich, St. Louis, MO, USA	RRID:AB_ 2057371
Bcas1	mouse	1:200	Santa Cruz; Dallas, TX, USA	RRID:AB_10839529
Clec7a (Dectin1)	rat	1:50	InvivoGen San Diego, CA, USA	RRID:AB_2753143
CD74	rat	1:200	Biolegend, San Diego, CA, USA	RRID:AB_2566502
Iba1	chicken	1:500	Aves Labs, Davis, CA, USA	RRID:AB_2910556
Tmem119	rabbit	1:100	Abcam, Cambridge, UK	RRID:AB_2921338
CD3	rabbit	1:400	Agilent, Santa Clara, CA, USA	RRID:AB_2335677
MBP	rat	1:300	Biorad, Hercules, CA, USA	RRID:AB_325004
